# Determinants of condom uptake among HIV voluntary counselling and testing clients: experiences from a hospital-based study in south India

**DOI:** 10.1186/1472-6963-12-13

**Published:** 2012-01-11

**Authors:** Rebecca Myerson, Susanna M Makela, C Chandrasekhar, Siju Mathew, Sourabh Chakraborty

**Affiliations:** 1 Institute for Health Metrics and Evaluation, Seattle, Washington, USA; 2 Government Hospital of Thoracic Medicine, Chennai, India; 3 International Training and Education Centre for Health (I-TECH) Arogyaan, Chennai, India

## Abstract

**Background:**

HIV voluntary counselling and testing was a key HIV prevention strategy brought to scale by India's National AIDS Control Organization. Condom uptake is an essential metric of intervention impact given the expansion of the epidemic into an increasingly diverse population. With only 20% of first-time counselling and testing clients at the largest HIV treatment hospital in south India reporting previous condom use, the question of intervention impact on condom use deserves investigation. In this study, we track intervention impact across various demographic groups and identify the added value of more thorough counselling.

**Methods:**

Data were collected from 8,865 individuals who attended counselling multiple times at the Tamil Nadu Government Hospital of Thoracic Medicine over the years 2004-2009. Counsellors recorded client demographic characteristics, HIV risk behaviours reported, and counselling services provided after each counselling session. Matching and regression methods were used to determine the probability of condom uptake by serostatus, gender, and receipt of personalized risk reduction counselling while controlling for other characteristics.

**Results:**

HIV counselling and testing was associated with condom uptake among 29.2% of HIV positive women (CI 24.5-34.4%), 31.7% of HIV positive men (CI 27.8-35.4%), 15.5% of HIV negative women (CI 11.2-20.8%), and only 3.6% of HIV negative men (CI 1.9-5.9%) who had previously never used condoms. Personalized risk reduction counselling increased impact in some groups; for example an additional 18% of HIV negative women (CI 11.3-24.4%) and 17% of HIV positive men (CI 10.9-23.4%) started using condoms. The number of sexual partners was not associated with the impact of counselling completeness.

**Conclusions:**

Because the components of testing and counselling impact the condom use habits of men and women differently, understanding the dynamics of condom use negotiation between partners is essential to optimizing impact on Indian couples. Clients' predicted condom uptake ranged between 4% and 47% depending on factors like gender, serostatus, and services provided. Personalized risk reduction counselling is associated with increased chance of condom use, with larger gains in HIV negative women and HIV positive men. HIV negative men are least likely to start using condoms and least impacted by additional counselling.

## Background

With approximately 2.4 million HIV cases in India and an adult prevalence of about 0.3%, prevention of new infections was of utmost importance in working toward the National AIDS Control Program Phase III (NACP-III) goal of halting and reversing the HIV/AIDS epidemic in India between 2006 and 2011 [1,2]. Monitoring and evaluation of prevention strategies and programs is essential.

Voluntary HIV counselling and testing (VCT) is one of the key NACP strategies for halting the HIV epidemic in India. NACP-III calls for 22 million clients to be counselled and tested in public Integrated Counselling and Testing Centres (ICTCs) each year [2]. VCT clients are given counselling relevant to their own personal risk profiles and informed of the purpose, benefits, and risks of an HIV test. After receiving their test results, clients also receive post-test counselling on how to prevent future infection or referrals to medical, social and psychological services depending on whether their test results are negative or positive.

Ideally, counselling services at VCT centres would facilitate primary prevention by altering clients' risky sexual behaviours; however, research findings are mixed regarding the effectiveness of testing and risk reduction counselling in reducing high risk behaviour. Some studies have linked HIV counselling and testing to significant increases in condom use [3,4], including increases of up to 50 percentage points [5], but these dramatic results are offset by other studies that show a much smaller effect [6], [7], [8]. This body of research indicates that the impact of counselling and testing on subsequent condom use may vary by individuals' and partners' serostatus, gender and risk group, with more successful results shown among serodiscordant couples [5], female sex workers [9,10] and truck drivers [11]; less successful results among HIV negative clients [12]; and mixed results among HIV positive men who have sex with men [13,14]. There is also evidence that gender moderates the relationship between HIV positivity and increased condom use [15].

Increases in condom use, as with any behaviour change, must be defined relative to a particular time frame. This study looks at changes in condom use over a period of one month or longer, which may seem very short for observing a true change in behaviour. While many studies have shown increases in condom use over a follow-up period of over 6 months [3], [4], [5], [10], [16], [17], [18], [19], several studies have also shown increases over shorter periods of time. Researchers have found increases in condom use among truck drivers after one to two months [11], men who have sex with men after 12 weeks [14], and Indian men and Americans visiting STD clinics [3,10] as well as intravenous drug users (IDUs) [20], after 6 months.

Previous studies in India have assessed the effectiveness of VCT on risk behaviour and HIV incidence for clients in key risk groups, such as men visiting STD clinics in western India [10] or sex workers in Bombay [21]. Less is known about the effectiveness of counselling on encouraging condom use outside those risk groups in India. Recent research indicates a rising HIV prevalence in married women, who are often placed at risk for HIV infection by the sexual behaviour of their husbands [22], [23], [24]. The international literature gives cause for optimism about the effectiveness of VCT services at increasing condom use among couples [4,12,19], but such results may not be replicated in an Indian population where gender roles, norms related to sexual practices, health beliefs and cultural beliefs all create significant barriers to condom uptake among married Indian men and women [25,26]. A recent study of condom use among women participating in a microbicide clinical trial in Pune, India, and their male partners showed increases in condom use by both groups at 2, 4, and 6 months after baseline [27]. This study is of particular importance as it provides evidence for the possibility of increased condom use among Indian couples who are not members of traditional high-risk groups. However, the study provided only condom counselling and HIV risk reduction counselling, not HIV testing and counselling as it would be delivered in a VCTC setting. Therefore, more research into completeness and effectiveness of counselling services delivered to clients outside previously studied risk groups is merited.

25 years after the first case of HIV was found in its capital of Chennai in 1986, the state of Tamil Nadu has become a leader in HIV prevention and treatment in India [28,29]. With its large client load and high profile, the testing and counselling service of the Government Hospital of Thoracic Medicine (GHTM) in Chennai represents a major government endeavour for primary and secondary prevention of HIV in Tamil Nadu. One of ten Centres of Excellence in HIV care in India and one of the largest HIV and AIDS care centres in South Asia, GHTM has enrolled over 8,600 patients in ART since 2004, and the testing centre currently serves an average of 90 (SD = 33) clients daily [30].

The distinction between effectiveness and efficacy is essential when assessing the impact of voluntary testing and counselling on clients' risk behaviours. Dropout or refusal of services may place clients out of reach of VCT counsellors, and cultural and sociological influences may prevent even the best counsellor from changing the behaviour of a client in two sessions. As we seek to measure the effectiveness of services in their typical delivery, our study uses observational data rather than data from a trial environment, and we track the completeness of services as well as predictors of condom uptake.

The purpose of this study is to assess the impact of testing and counselling services at the GHTM on condom uptake by gender, history of multiple sex partners and serostatus, focusing on clients who reported not using condoms at baseline and returned to the ICTC multiple times over a series of months. In contrast to previous studies, we focus on individuals who do not belong to traditional higher risk groups such as men who have sex with men, commercial sex workers, or truck drivers, as we wish to examine the effects of risk-reduction counselling clients belonging to the larger VCT population. Additionally, we recognize that not all clients received the full intervention, and we analyse the intervention as delivered in more complete and less complete versions. This research, paired with future studies probing the societal, psychological and logistical factors involved, can help staff at HIV counselling and testing centres in India identify best practices for strategic use of funds and ensure maximum impact on the HIV and AIDS epidemic.

## Methods

### Study population and procedures

Data were collected from the Integrated Counselling and Testing Centre (ICTC) at the Government Hospital of Thoracic Medicine in Chennai, India from 2004-2009. At the centre, clients were given counselling relevant to their own personal risk profiles and informed of the purpose, benefits, and risks of an HIV test. After receiving their test results, clients also received post-test counselling explaining the implications of their test results and how to prevent future infection or referrals to medical, social, and psychological services depending on whether their test results were negative or positive. Because sex and sexuality are taboo subjects in India, counsellors worked to develop a rapport with clients to gather accurate information, and did not take notes during interviews.

Although all clients should have received personalized risk reduction counselling according to national guidelines, only 67.7% of clients received such services in practice. Because almost all HIV in this region is transmitted heterosexually [22], we assumed that risk reduction counselling would include discussion of condoms.

### Data

Primary data was used in the study, in the form of questionnaires completed by the counsellors after each counselling session. Standard hospital protocol required that after each visit with a client, counsellors circled items on a multiple choice questionnaire to indicate which counselling services they had provided and what they had learned about the client's background and risk behaviours. This took place as soon as possible after each visit, so that data were collected prospectively. Patients' ICTC records were not linked with their medical records with the exception of HIV status, which was analysed by the GHTM laboratory and merged using patients' unique ID number. HIV status was established using ELISA and was recorded even for patients who never returned to retrieve their results. The dataset used for analysis was stripped of personal identifiers. The GHTM internal review board gave ethical approval for the study and granted access to the data.

Among new clients attending the testing centre between 2004 and 2009, 8,865 subsequently returned at least one month after their first visit, enabling calculation of changes in reported condom use. Clients who returned were more likely to be HIV positive than those who did not, but the two groups were within a 5-7% range on other demographic factors. ^1^See Table 1 for a summary of demographic factors.

**Table 1:** Demographics by receipt of a second visit

	One visit			Two or more visits		
**Characteristic**	**N**	**%**	**HIV prev.*^a^***	**N**	**%**	**HIV prev.*^a^***

**Total**	94020		34.1	8865		58.6
**Sex**						
**Female**	35083	37.3%	38.6	3486	39.3%	68.3
**Male**	58937	62.7%	31.5	5379	60.7%	52.3
**Age**						
**15-19**	1784	1.9%	16.3	72	0.8%	44.4
**20-29**	22433	23.9%	46.7	2336	26.4%	74.1
**30-39**	28396	30.2%	48.8	3245	36.6%	71.8
**40-49**	18901	20.1%	29.4	1727	19.5%	48.9
**50+**	22506	23.9%	8.5	1485	16.8%	17.2
**Education**						
**None/primary**	61528	65.4%	30.4	5105	57.6%	54.5
**Secondary/graduate**	32492	34.6%	41.2	3760	42.4%	64.1
**Current marital status**						
**Married**	74800	79.6%	33.9	7502	84.6%	57.7
**Unmarried**	19220	20.4%	34.9	1363	15.4%	63.6
**Occupation**						
**Housewife**	15974	17.0%	39.2	1763	19.9%	64.9
**Other/unemployed**	9006	9.6%	12.9	579	6.5%	21.6
**Professional/manager**	2642	2.8%	48.5	260	2.9%	69.2
**Skilled**	35733	38.0%	40.2	3625	40.9%	65.8
**Student**	723	0.8%	5.4	26	0.3%	23.1
**Unskilled**	29942	31.8%	30	2612	29.5%	51.8
**Lifetime sexual partners**						
**0 or 1**	57283	60.9%	23.5	4898	55.3%	46.4
**2 or more**	36737	39.1%	50.8	3967	44.7%	73.6

*^a ^*HIV prevalence per 100 clients.

**Figure 1:**
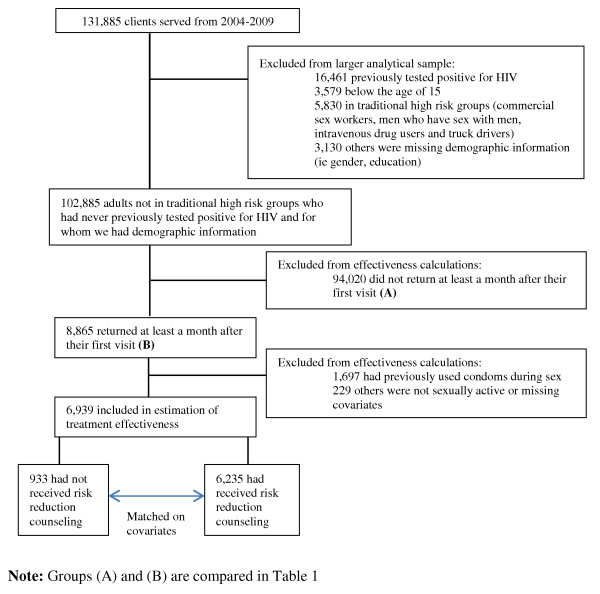
**Flow chart of data selection and processing**.

See Figure 1 for a flow chart depicting sample selection and processing of the data. To investigate the determinants of condom uptake with or without risk reduction counselling, we analysed data from a subset of 6,939 clients who had never used condoms before their first visit, were sexually active, and had information on all covariates of interest. We considered clients to have received more complete counselling (including personalized risk reduction counselling) if counsellors circled items on the first visit post-counselling questionnaire indicating that they discussed and recommended condom use to the client or discussed a personalized risk reduction plan. In addition, we considered clients to have started using condoms if the counsellor recorded condom use on any subsequent visit at least one month after the client's original visit.

### Statistical analysis

As these data are not from a randomized trial, we anticipated that delivery of personalized risk reduction counselling might depend on clients' backgrounds. For this reason, we used matching and regression methods to ensure an identical covariate distribution for the two counselling completeness groups and to control for additional confounding.

First, we used a non-parametric matching procedure to achieve identical covariate distributions in the two treatment groups. Table 2 summarizes this procedure. Clients who received risk reduction counselling or discussed condom use with a counsellor were matched with clients who did not receive these services but reported identical age group (15-34, 35+), education (less than secondary, secondary or more), HIV serostatus, total number of previous and current sex partners reported at first visit (0 or 1, 2 or more), current marital status (married, unmarried), and gender. Matches were found for 6,929 observations (99.9%). In the input data, 67.7% of clients had received more complete counselling and 32.3% had not, but weights were used to assign equal importance to clients who had received more complete vs. less complete counselling in the subsequent analysis [31].

**Table 2:** Review of matching procedure used

	Less complete vs. more complete VCT
**Purpose of matching**	Prepare data to calculate the difference in condom uptake expected after less complete vs. more complete VCT
**Matching strategy**	Exact matching
**Variables matched in each pair:**	
**Age group**	15-34, 35+
**Education**	Less than secondary education, secondary or more
**HIV status**	HIV positive, HIV negative
**Gender**	Male, female
**Other factors**	Current marital status, history of condom use (all had not used condoms before)
**Variable that differed in each pair:**	Completeness of VCT received
**Outcome of interest**	Condom uptake

After matching treated and untreated clients by age group, education, HIV serostatus, history of multiple partners, current marital status and gender, we used a logistic regression to facilitate predictions of clients' probability of condom uptake while controlling for demographic and behavioural factors. Trends in the raw data indicated three-way interactions between receipt of risk reduction counselling, sex, and HIV status, as well as between counselling, sex, and history of multiple partners in predicting condom uptake. For this reason, we included these three-way interaction terms in the regression, whereas two-way interactions were included for all other variables. Stata dropped some higher-order terms due to collinearity. Due to the large number of interaction terms, coefficients are presented rather than odds ratios to encourage the reader to consider the whole model. See Table 3. After running the logistic regression, we used simulation modelling (1,000 simulations per scenario) to create predicted results for hypothetical sexually active clients by serostatus, history of multiple sex partners and gender. Predicted condom uptake was calculated for each hypothetical client under scenarios of more complete and less complete counselling. Hypothetical clients were assigned the mean frequency for their gender for the characteristics of interest: positive HIV serostatus (33.2% and 41.3% for men and women, respectively), secondary education or higher (37.6% and 31.3%), age 35 or older (67.8% and 43.6%), and multiple partners (55.9% and 12.3%) [32]. Results are shown in Table 4.

**Table 3:** Logistic regression coefficients

	Mean	Lower CI		Upper CI
***Main effects:***				
**Currently married**	1.11	(0.75	-	1.46)
**Male**	-1.06	(-1.47	-	-0.68)
**Age 35+**	-0.5	(-0.73	-	-0.26)
**Secondary education**	0.48	(0.27	-	0.69)
**HIV positive**	0.94	(0.52	-	1.34)
**Multiple sexual partners**	-0.19	(-0.73	-	0.34)
**Received risk reduction counseling ("treated")**	1.33	(0.78	-	1.9)
**Constant**	-2.73	(-3.25	-	-2.21)
***Interaction terms:***				
**Currently married, treated**	0.17	(-0.24	-	0.62)
**Age 35+, treated**	-0.08	(-0.34	-	0.2)
**Secondary education, treated**	-0.22	(-0.47	-	0.04)
**HIV negative male, treated**	-1.26	(-1.76	-	-0.78)
**HIV positive female, treated**	-0.75	(-1.22	-	-0.28)
**HIV positive male, untreated**	1.51	(0.73	-	2.28)
**0 or 1 sexual partners, female, untreated**	-0.44	(-0.95	-	0.1)
**0 or 1 sexual partners, male, untreated**	-0.89	(-1.51	-	-0.23)
**Multiple sexual partners, male, treated**	0.87	(0.31	-	1.4)
**Multiple sexual partners, female, untreated**	-0.01	(-0.64	-	0.68)

Note: N = 6,939. STATA automatically dropped some third order interaction terms due to collinearity

**Table 4:** Predicted probability (%) of condom uptake among clients who had never used a condom before attending the ICTC, using model based correction vs.raw data

	Less Complete VCT Services (No Risk Reduction Counseling)		More Complete VCT Services (With Risk Reduction Counseling)	
	**Mean**	**95% CI**	**Mean**	**95% CI**

**Model based correction**				
**HIV + Man**	29.2	(24.5-34.4)	46.5	(43.1-49.9)
**HIV + Woman**	31.7	(27.8-35.4)	37.8	(35.3-40.3)
**HIV - Man**	3.6	(1.9-5.9)	8.9	(7-11.1)
**HIV - Woman**	15.5	(11.2-20.8)	33.5	(29.1-38.3)
**Man, 0 or 1 partners**	5.3	(3-8.1)	12.2	(9.7-15.3)
**Man, Multiple Partners**	10	(6.5-14.1)	21.4	(18.3-24.8)
**Woman, 0 or 1 partners**	21.6	(17.8-25.4)	34.6	(31.6-37.6)
**Woman, Multiple Partners**	18.7	(12.6-26.1)	40.2	(34.4-46.5)
**Raw data**				
**HIV + Man**	42.2	(37.3-47.2)	57	(54.7-59.2)
**HIV + Woman**	40.7	(35.3-46.2)	45.1	(42.6-47.6)
**HIV - Man**	3.4	(2.4-4.6)	8.8	(7.1-10.8)
**HIV - Woman**	19.6	(15.9-23.8)	41.4	(37.1-45.8)
**Man, 0 or 1 partners**	5.1	(3.8-6.7)	13.2	(11.1-15.5)
**Man, Multiple Partners**	21.5	(18.9-24.3)	48.1	(46.1-50.1)
**Woman, 0 or 1 partners**	23.9	(21.1-27)	38.8	(36.7-40.9)
**Woman, Multiple Partners**	22.8	(15.5-31.6)	38.3	(33.6-43.1)

Note: N = 6,939.

**Figure 2:**
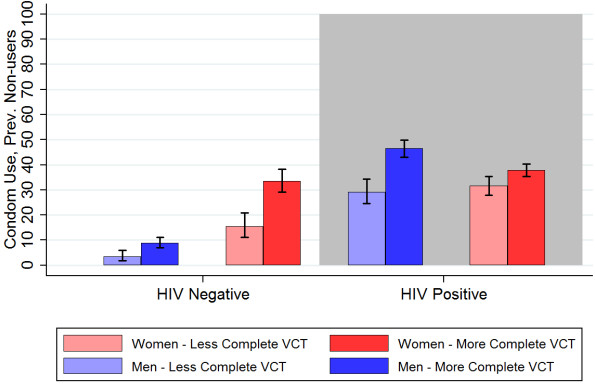
**Predicted probability of condom uptake among clients who had never used a condom before, by sex, HIV status, and receipt of risk reduction counselling**.

The relevance of including the interaction terms in the logistic regression, as shown in Table 3, can be seen in the simulated results for different client types in Table 4. For example, the model-predicted results capture the moderating effects of gender on the relationship between HIV status and condom uptake: men with HIV were much more likely than men without HIV to start using condoms, whereas this gap was smaller for women. These moderating effects extended to estimation of the additional effect of more thorough counselling. See Table 4 and Figure 2.

Analysis was conducted using Stata 10 and R 2.11.1. The MatchIt package in R was used for the matching procedure.

## Results

Table 4 shows predicted probability of condom uptake for hypothetical sexually active clients by gender, serostatus, education and history of multiple partners. The model-corrected estimates, some of which are also depicted in Figure 2, show more modest and consistent treatment effects than the raw data, with all client groups showing higher predicted condom uptake with risk reduction counselling than without risk reduction counselling.

We estimated that 15.5% of HIV negative women (CI 11.2-20.8%) and only 3.6% of HIV negative men (CI 1.9-5.9%) who had never used condoms before attending the ICTC reported starting to use condoms after receiving less complete VCT, that is, without risk reduction counselling. In contrast, 33.5% (CI 29.1-38.3%) of HIV negative women or 8.9% (CI 7-11.1%) of HIV negative men reported starting to use condoms after receiving the fuller quality intervention. HIV positive clients were more likely than negative clients to report starting to use condoms even without complete counselling, and uptake was similar for positive men (31.7%, CI 27.8-35.4%) and positive women (29.2%, CI 24.5-34.4%). Receipt of more complete counselling was associated with an additional increase of 17.2% (CI 10.9-23.4%) for HIV positive men and an additional increase of 6.1% (CI 1.5-10.7%) for HIV positive women. We did not detect a significant difference in treatment effect by number of previous sexual partners.

## Discussion

In this analysis, we looked beyond mean effects to estimate the impact of the intervention in various subgroups. Gender and serostatus played a major role in defining clients' chances of condom uptake, which varied between 9% and 47% even after receipt of more complete counselling. This wide variation is nevertheless in line with several studies examining condom uptake after counselling and testing in India among men and women who are not members of high-risk groups (i.e., commercial sex workers, truck drivers, and IDUs) [10,27]. Of all subgroups considered in the analysis, HIV positive men were the most likely to start using condoms after receiving risk reduction counselling, while HIV negative men were the least likely to do so. This is concerning because men were more likely than women to have a history of multiple sex partners: 12.3% of women in the ICTC dataset had a history of multiple sex partners (CI 11.9%-12.7%) in contrast with 55.9% of men (CI 55.5%-56.2%). If the HIV negative men were to contract HIV from a non-marital sex partner, their wives would also be placed at risk for HIV infection as well, a pattern that has led to an increase in HIV prevalence among married monogamous Indian women [22], [23], [24]. Furthermore, Indian men may override their wives' preferences for condom use due to the power dynamics of their marriages [25,26].

We did not find significant differences in treatment impact by history of multiple partners, although non-significant trends indicated that individuals with multiple partners may be more likely than others to start using condoms after VCT. Previous studies have shown that IDUs with multiple sex partners are more likely to increase condom use post-intervention [20].

We also examined the impact of VCT under multiple quality scenarios. Only two-thirds of clients at the GHTM received the full intervention content including pre-test counselling, post-test counselling, and personalized risk reduction counselling, and we found an association between more complete services and increased intervention impact in all subgroups considered. Completeness of counselling in terms of personalized risk reduction counselling had the largest impact on condom uptake among HIV negative women and HIV positive men. Other aspects of counselling completeness may have implications for treatment impact in India: in a parallel analysis, we have estimated that if clients learned to use condoms with maximum effectiveness (95% protective against HIV) rather than minimal effectiveness (70% protective against HIV), this could lead to a 25% decrease in the number of clients needed to test and counsel in order to avoid an HIV infection within a year [33].

While many studies on the effectiveness of VCT focus on client uptake of condom use [3], [4], [5], [6], [10], [19], other studies interviewing counsellors themselves indicate that using increases in condom use as the sole metric of VCT effectiveness and success may not give an accurate picture of the benefits conferred to clients. As illustrated by the present study, achieving behaviour change can be very difficult, particularly in the context of the numerous cultural barriers faced by clients in countries like India. As even the best counsellor cannot always induce behaviour change [34], metrics of counselling success and effectiveness based solely on behaviour change metrics may yield misleading results as to the value and quality of VCT.

For this reason, our results should be interpreted in the context of effective coverage, where the quality of services is benchmarked as a fraction of the maximum possible treatment effect [35]. We cannot expect that any type of counselling could cause 100% of clients to start using condoms. Rather, the maximum possible treatment effects for Indian clients of the gender, serostatus and sexual risk groups explored in this study lie somewhere between the observed treatment effects and 100% condom uptake.

### Strengths

This study has several important strengths. The data used for this study represent one of the largest samples of counselling and testing centre clients in India. The HIV prevalence of the clients was high, giving us power to detect differences in condom uptake between HIV negative and positive clients by gender and sexual history. GHTM is a certified centre of excellence in India and its laboratories meet American CDC standards, yielding HIV lab results that are reliable and valid. Lab results were complete regardless of whether clients return to get their results. In addition, data were collected prospectively in the form of notes taken after each counselling visit, helping to minimize recall bias on the part of the counsellor if not on the part of the client.

Although we used observational data, we used both matching and regression techniques to control for confounding. We also made explicit our goal of measuring effectiveness in the real-world setting, rather than efficacy in an ideal setting, thereby using the data on incomplete treatment as an advantage.

### Limitations

All of the data about sexual history and behaviour are self-reported. Such data are subject to recall bias. Furthermore, talking about sex and sexuality is taboo in India, and many clients may have felt reluctant to give accurate answers to many of the questions counsellors ask during a session.

Although only one quality metric was analysed in this paper, we would ideally like to expand data collection to include additional quality metrics. For example, counsellors at GHTM are instructed to demonstrate correct condom use but we have no data verifying that this is actually done; in a population with generally low levels of condom knowledge, demonstrations may be necessary as a part of effective counselling, and condom uptake with incorrect use may not provide adequate protection from HIV. In addition, counsellors may vary in their knowledge about HIV or prevention instruction experience; this would result in and inconsistent intervention quality, a sort of information bias.

In our study, we chose to analyse the effect of risk reduction counselling on condom uptake only among clients who returned to the counselling and testing centre over a month after their original visit. Readers may question whether one month is a sufficiently long period of time to expect behaviour change. In fact, several studies have observed that increases in condom use during an intervention are not sustained once the study is over [27,36], indicating that shorter time frames may give a more accurate measurement of the true intervention effect. This time restriction led to a substantial reduction in the size of our dataset, but Table 1 indicates minor differences between the subsample and larger population in terms of gender, education, age, marital status, occupation and number of sexual partners. Despite the limitations posed by time frame restrictions in this study, previous studies have detected increases in condom use over similar intervals, among both high-risk and non-high-risk individuals. In previous research, female microbicide trial participants and their male partners showed increases in condom use among two months after baseline [27], truck drivers in China exhibited increased condom use in follow-up surveys one and two months after baseline [11], and men who have sex with men increased their condom use 12 weeks after baseline [14].

## Conclusions

To our knowledge, this is the first study to examine the differential effects of more complete and less complete HIV counselling and testing on condom uptake in India. Importantly, we focus on ICTC clients who are outside of traditional Indian risk groups, allowing us to examine the potential effects of counselling on members of the larger population.

Our results indicate that completeness of counselling had the largest impact on condom uptake among HIV negative women and HIV positive men. Non-significant trends indicate a possible boost in condom use after receipt of personalized risk reduction counselling among individuals with a history of multiple partners. These results suggest that more thorough counselling may be a particularly good investment for members of serodiscordant couples where the man is HIV positive and for members of couples in which one partner has multiple sex partners, and it could potentially play a role in slowing the rising prevalence of HIV among married monogamous women. Further research is needed to determine whether the low observed condom use among HIV-negative men is due to a lower maximum possible treatment effect for these men in the Indian context or due to an ineffective counselling strategy.

## Competing interests

The authors declare that they have no competing interests.

## Authors' contributions

RM and SMM conducted the analysis, drafted the manuscript and conducted revisions. CC conceived of the study and arranged access to data. SM and SC participated in design and coordination of the analysis and helped to edit the manuscript. All authors read and approved the final manuscript.

## Authors' information

At the time the research was conducted, Rebecca Myerson and Susanna Makela were affiliated with the Institute for Health Metrics and Evaluation at the University of Washington in Seattle, Washington, and Siju Mathew and Sourabh Chakraborty were affiliated with the International Training and Education Centre for Health (I-TECH) Arogyaan in Chennai, India. Rebecca Myerson is currently a PhD student at the University of Chicago, Susanna Makela is a research fellow at the Public Health Foundation of India in Delhi, Siju Mathew is a research associate at the Institute for Financial Management and Research in Chennai, and Sourabh Chakraborty is affiliated with the Kusuma Foundation in Delhi.

## Pre-publication history

The pre-publication history for this paper can be accessed here:

http://www.biomedcentral.com/1472-6963/12/13/prepub

## Endnotes

^1^Results indicate that the subsample (58.6%, CI 57.6-59.6%) had higher HIV prevalence than the remainder of the sample (34.1%, CI 33.8%-34.4%, χ2 (1) = 2100, p < 0.001). Although the subsample was more educated, more likely to be married and more likely to have had multiple partners, these differences were minor: 57.5% (56.5%-58.6%) of clients in the subsample had not received secondary education, versus 65.4% (65.1%-65.7%) in the rest of the sample; 84.6% (83.9%-85.3%) of clients in the subsample were married, compared with 79.6% (79.2-79.8%) of the rest of the sample; 44.7% (43.7-45.8%) of clients in the subsample had had more than one sexual partner, compared with 39.1% (38.8-39.4%) in the remaining sample. Differences in gender were even less substantial: 39.3% (CI 38.3-40.3%) of the subsample were female, versus 37.3% (37%-37.6%) of the remainder of the sample. Chi squared tests comparing the two samples on all these variables were significant.

## References

[B1] UNAIDS Epidemiological Fact Sheet on HIV and AIDS: India 2009 UNAIDS/WHO Working Group on Global HIV/AIDS and STI Surveillance

[B2] National AIDS Control Program - Phase III (2009-2011): strategy and implementation plan 2007 Ministry of Health and Welfare, Government of India

[B3] Kamb M Fishbein M Douglas J Rhodes F Rogers J Bolan G Zenilman J Hoxworth T Malotte K Iatesta M Efficacy of risk reduction counseling to prevent Human Immunodeficiency Virus and sexually transmitted diseases: a randomized controlled trial JAMA 1998 280 1161 1167 10.1001/jama.280.13.1161 9777816

[B4] Allen S Meinzen-Derr J M K Zulu I Trask S Fideli U Musonda R Kasolo F Gao F Haworth A Sexual behavior of HIV discordant couples after HIV counselling and testing AIDS 2003 17 733 740 10.1097/00002030-200303280-00012 12646797

[B5] Allen S Tice J Van de Perre P Serufilira A Hudes E Nsengumuremyi F Bogaerts J Lindan C Hulley S Effect of serotesting with counselling on condom use and seroconversion among HIV discordant couples in Africa British Medical Journal 1992 304 1605 1609 10.1136/bmj.304.6842.1605 1628088 PMC1881972

[B6] Xu F Kilmarx P Supawitkul S Manopaiboon C Yanpaisarn S Limpakarnjanarat K Chaikummao S Mock P Young N Mastro T Incidence of HIV-1 infection and effects of clinic-based counselling on HIV preventive behaviours among married women in northern Thailand Journal of AIDS 2002 29 284 288 11873078 10.1097/00042560-200203010-00009

[B7] Kawichai S Beyrer C Khamboonruang C Celentano D Natpratan C Rungruengthanakit K Nelson K HIV incidence and risk behaviours after voluntary HIV counselling and testing (VCT) among adults aged 19-35 years living in peri-urban communities around Chiang Mai city in northern Thailand, 1999 AIDS Care 2004 16 21 35 10.1080/09540120310001633949 14660141

[B8] Denison J O'Reilly K Schmid G Kennedy C Sweat M HIV voluntary counselling and testing and behavioural risk reduction in developing countries: a meta-analysis 1990-2005 AIDS and Behavior 2008 12 363 373 10.1007/s10461-007-9349-x 18161018

[B9] Shahmanesh M Patel V Mabey D Cowan F Effectiveness of interventions for the prevention of HIV and other sexually transmitted infections in female sex workers in resource poor settings: a systematic review Tropical Medicine & International Health 2008 13 659 679 10.1111/j.1365-3156.2008.02040.x 18266784

[B10] Bentley M Spratt K Shepherd M Gangakhedkar R Thilikavathi S Bollinger R Mehendale S HIV testing and counselling among men attending sexually transmitted disease clinics in Pune India: changes in condom use and sexual behavior over time AIDS 1998 12 1869 1877 10.1097/00002030-199814000-00019 9792388

[B11] Lau J Tsui H Cheng S Pang M A randomized controlled trial to evaluate the relative efficacy of adding voluntary counselling and testing (VCT) to information dissemination in reducing HIV-related risk behaviours among Hong Kong male cross-border truck drivers AIDS Care 2010 22 17 28 10.1080/09540120903012619 20390477

[B12] Weinhardt L Cary M Johnson B Bickman N Effects of HIV counselling and testing on sexual risk behavior: A meta-analytic review of published research 1985-1997 American Journal of Public Health 1999 89 1397 1405 10.2105/AJPH.89.9.1397 10474559 PMC1508752

[B13] Darrow W Webster R Kurtz S Buckley A Patel K Stempel R Impact of HIV counselling and testing on HIV-infected men who have sex with men: the South Beach health survey AIDS and Behavior 1998 2 115 126 10.1023/A:1022142812952

[B14] Fox J White P Macdonald N Weber J McClure M Fidler S Ward H Reductions in HIV transmission risk behaviour following diagnosis of primary HIV infection: a cohort of high-risk men who have sex with men HIV Medicine 2009 10 432 438 10.1111/j.1468-1293.2009.00708.x 19459996

[B15] Huchko M Montandon M Nguti R Bukusi E Cohen C The association of HIV counselling and testing with HIV risk behaviours in a random population based survey in Kisumu, Kenya AIDS and Behavior 2011 15 718 724 10.1007/s10461-009-9649-4 20012479 PMC2997911

[B16] Cornman D Schmiege S Bryan A Benziger T Fisher J An information-motivation-behavioral skills (IMB) model-based HIV prevention intervention for truck drivers in India Social Science and Medicine 2007 64 1572 1584 10.1016/j.socscimed.2006.11.011 17257724 PMC4675654

[B17] Turner A Miller W Padian N Kaufman J Behets F Chipato T Mmiro F Salata R Morrison C Unprotected sex following HIV testing among women in Uganda and Zimbabwe: short- and long-term comparisons with pre-test behaviour International Journal of Epidemiology 2009 38 997 1007 10.1093/ije/dyp171 19349481 PMC2720394

[B18] Arthur G Nduba V Forsythe S Mutemi R Odhiambo J Gilks C Behaviour change in clients of health centre-based voluntary HIV counselling and testing services in Kenya Sexually Transmitted Infections 2007 83 541 546 10.1136/sti.2007.026732 17991688 PMC2598642

[B19] Efficacy of voluntary HIV-1 counselling and testing in individuals and couples in Kenya Tanzania and Trinidad: a randomized trial. Lancet 2004 356 103 112 10963246

[B20] Kwiatkowski C Stober D Booth R Zhang Y Predictors of increased condom use following HIV intervention with heterosexually active drug users Drug and Alcohol Dependence 1999 54 57 62 10.1016/S0376-8716(98)00145-8 10101617

[B21] Bhave G Lindan C Hudes E Desai S Wagle U Tripathi S Mandel J Impact of an intervention on HIV, sexually transmitted diseases and condom use among sex workers in Bombay, India AIDS 1995 9 S21 S30 8561997

[B22] Solomon S Chakraborty A Yepthomi R A review of the HIV epidemic in India AIDS Education and Prevention 2004 16 155 169 10.1521/aeap.16.3.5.155.35534 15262573

[B23] Gangakhedkar R Bentley M Divekar A Gadkari D Mehendale S Shepherd M Bollinger R Quinn T Spread of HIV infection among married monogamous women in India JAMA 1997 278 2090 2092 10.1001/jama.1997.03550230066039 9403424

[B24] Newmann S Sarin P Kumarasamy N Amalraj E Rogers M Madhivanan P Flanigan T Cu-Uvin S McGarvey S Mayer K Solomon S Marriage, monogamy and HIV: a profile of HIV infected women in south India International Journal of STDs and AIDS 2000 11 250 253 10.1258/0956462001915796 10772089

[B25] Go V Sethulakshmi C Bentley M Sivaram S Srikrishnan A Solomon S Celentano D When HIV prevention messages and gender norms clash: The impact of domestic violence on women's HIV risk in slums in Chennai, India AIDS and Behavior 2003 7 263 272 10.1023/A:1025443719490 14586189

[B26] Bhattacharya G Sociocultural and behavioural contexts of condom use in heterosexual married couples in India: challenges to the HIV prevention program Health Education and Behavior 2004 31 101 117 10.1177/1090198103259204 14768661

[B27] Marlow H Tolley E Weaver M Kohli R Mehendale S Changes in condom use during a microbicide clinical trial in Pune, India AIDS Care 2011 10.1080/09540121.2011.630343 22088145

[B28] John T Babu P Jayakumari H Simoes E Prevalence of HIV infection in risk groups in Tamil Nadu, India Lancet 1987 1 160 161 2879994 10.1016/s0140-6736(87)91992-1

[B29] Solomon S Ganesh A Ekstrand M Barclay J Kumarasamy N Mandel J Lindan C High HIV seropositivity at an anonymous testing site in Chennai, India: client profile and trends over time AIDS and Behavior 2000 4 71 81 10.1023/A:1009540908691

[B30] I-TECH country program profile: India Available online at http://www.go2itech.org/where-we-work/india/handouts/IndiaProfile031108.pdf/view

[B31] Ho D Imai K King G Stewart E Matching as nonparametric preprocessing for reducing model dependence in parametric causal inference Political Analysis 2007 15 199 236

[B32] King G Tomz M Wittenberg J Making the most of statistical analyses: improving interpretation and presentation American Journal of Political Science 2000 341 355

[B33] Myerson R Effectiveness of counselling and testing for prevention of HIV infections by promoting condom use in Tamil Nadu, India 2011 Masters Thesis. University of Washington

[B34] Grinstead O Van der Straten A Voluntary HIV-1 Counseling and Testing Efficacy Study Group Counselors' perspectives on the experience of providing HIV counselling in Kenya and Tanzania: the voluntary HIV-1 counselling and testing efficacy study AIDS Care 2000 12 625 642 10.1080/095401200750003806 11218548

[B35] Shengelia B Tandon A Adams O Murray C Access utilization quality and effective coverage: an integrated conceptual framework and measurement strategy Social Science and Medicine 2005 61 97 109 10.1016/j.socscimed.2004.11.055 15847965

[B36] van der Straten A Cheng H Minnis A Change in condom and other barrier method used during and after an HIV prevention trial in Zimbabwe Journal of the International AIDS Society 2010 13 39 10.1186/1758-2652-13-39 20955629 PMC2984577

